# Factors of abandonment of tuberculosis treatment in the public health network

**DOI:** 10.11606/s1518-8787.2023057004454

**Published:** 2023-03-15

**Authors:** Claudia Lorena Perlaza, Freiser Eceomo Cruz Mosquera, Luisa Maria Ramirez Murillo, Valentina Becerra Sepulveda, Cindy Dayan Córdoba Arenas

**Affiliations:** I Universidad Santiago de Cali Facultad de Salud Cali Colombia Universidad Santiago de Cali. Facultad de Salud. Cali, Colombia

**Keywords:** Tuberculosis, prevention & control, Treatment Refusal, Antitubercular Agents, supply & distribution, Barriers to Access of Health Services

## Abstract

This study determines the factors of abandonment of tuberculosis treatment in the public health network of Cali, Colombia, during years 2016 to 2018. We conducted an operational case-control investigation including 224 patients with tuberculosis (112 abandoned treatment and 112 completed it). We found that treatment abandonment for tuberculosis is driven by factors related to the individuals and health services that facilitate non-adherence and drive them away from the care provided in medical institutions.

## INTRODUCTION

Tuberculosis is one of the ten leading causes of death in the world, caused by Mycobacterium tuberculosis, and affects all age groups. In 2019, there were 10 million cases, with 1.4 million deaths. This disease, with timely diagnosis and treatment in accordance with the international framework for tuberculosis control, guarantees survival for those affected, with a treatment success rate of 57% at world level^1^.

Multidrug-resistant tuberculosis, resulting from drug reactions and loss of follow-up treatment, accounts for half a million cases around the world. Interrupted administration of the drug is a determining factor in this resistance, causing a threat to public health and the health system, blocking the initiative to put an end to tuberculosis worldwide, and to the achievement of the Sustainable Development Goals, in which a 90% coverage of treatment is expected in 2023^1^. Colombia, as a country, has a high disease burden of 19,000 cases of tuberculosis per year, and an incidence rate of 35.8 per 100,000 inhabitants^2^. Therefore, this study aims to determine the factors of abandonment of tuberculosis treatment in the public network in Cali, Colombia, which is responsible for the care of the poor and vulnerable population, in a city that has a mortality rate above the national rate and increasing tuberculosis mortality in the world^1,2^.

## METHODS

Operational research with case-control design, carried out in the public health network of Santiago de Cali, with data provided by the tuberculosis program of the Municipal Public Health Secretariat. We included patients over 18 years old, residents of Cali, with a diagnosis of tuberculosis, who entered the program between January 1, 2016 and December 31, 2018, and presented a condition of abandonment of treatment or loss to follow-up. Abandonment was considered as not having started treatment or interruption of treatment for a period equal to or greater than one month.

We used the formula for estimating proportions to calculate the sample size, considering: n = 721 subjects who abandoned treatment during the study period, confidence level of 95%, precision of 5% and expected proportion of 10% according to data from the Municipal Public Health Secretariat of Cali, which suggested a minimum sample of 112 people. Participants were selected from simple random probability sampling and were compared with 112 subjects who completed the treatment.

The study variables included sociodemographic variables such as age, sex, ethnicity, municipality of residence and health system affiliation, as well as clinical factors such as HIV-positive diagnosis, diabetes, malnutrition, smoking, alcoholism and drug dependence. Finally, factors inherent to the health service were considered, such as bacteriological controls performed, months of treatment received, centralized tuberculosis program and multidisciplinary health team.

Data analysis was performed using SPSS version 24 software. We used the Kolmogorov Smirnov test to determine the normality of the variables. Categorical variables were expressed as frequency and percentage and quantitative variables as mean plus standard deviation. In the univariate analysis, we used chi-square for differences in proportions and Student’s t-test for differences in means, assuming a p-value < 0.05 as statistically significant. In the bivariate analysis, to establish the crude association between the independent variables and tuberculosis treatment abandonment, we used contingency tables from which odds ratios with their respective 95% confidence intervals were estimated. Finally, we performed a binary logistic regression with Hosmer-Lemeshow Goodness of Fit and backward stepwise selection method, considering as elimination criterion the variables with a significance greater than 0.20.

The research followed the ethical guidelines of Ministry of Health Decision 8430 and the Helsinki Declaration. The protocol was ethically endorsed by the Universidad Santiago de Cali and authorized by the Secretary of Public Health of the Municipality of Cali.

## RESULTS

We studied 224 subjects homogeneously distributed in the tuberculosis treatment abandonment and non-abandonment groups. The majority were male (70%), racially mixed (81%) and (56%) under 40 years old. Regarding affiliation to the General Social Security Health System, 77% were in the subsidized system and the rest were not affiliated. Finally, in the binary logistic regression model, factors associated with treatment abandonment were identified as male sex (OR = 2. 2; 95%CI 1.1-4.5) age under 40 years (OR = 2.2; 95%CI 1.2-4.2) being part of a vulnerable population (OR = 3; 95%CI 1.6-5.8) not being insured (OR = 5.3; 95%CI 2.2-12.3) and not having been tested for HIV (OR = 5.1; 95%CI 1.5-17.8) (Table).

**Table:** Factors associated with abandonment of tuberculosis treatment.

Characteristic	Abandonment n = 112	No Abandonment n = 112	p	Crude OR	95%CI	Exp (B)	95%CI
	
	n (%)	n (%)					
Sex							
Man	86 (77)	70 (63)	0.02	1.9	1.1–3.5	2.2	1.1–4.5
Woman	26 (23)	42 (37)					
Age							
< 40 years old	77 (69)	49 (44)	0.001	2.8	1.6–4.8	2.2	1.2–4.2
> 40 years old	35 (31)	63 (56)					
Ethnicity							
Mixed race	95 (85)	88 (79)	0.22	1.5	0.7–2.0		
Other	17 (15)	24 (21)					
Type of population							
Vulnerable	63 (56)	25 (22)	0.0001	4.4	2.5–7.9	3	1.6–5.8
Other	49 (44)	85 (78)					
SGSSS affiliation system							
Uninsured	41 (37)	10 (9)	0.0001	5.8	2.7–12.5	5.3	2.2–12.3
Subsidized	71 (63)	102 (91)					
Centralized ESE							
No	38 (34)	40 (36)	0.7	1.08	0.6–1.8		
Yes	74 (66)	72 (64)					
Bacilloscopy diagnostic							
Negative	8 (7)	4 (4)	0.4				
One cross	30 (27)	38 (34)					
Two crosses	36 (32)	31 (27)					
Three crosses	38 (34)	39 (35)					
HIV test							
No	15 (13)	5 (4)	0.019	3.3	1.1–9.4	5.1	1.5–17.8
Yes	97 (87)	107 (96)					
Pathological history							
HIV	15 (13)	9 (8)	0.1	1.7	0.7–4.2		
Malnutrition	16 (14)	19 (17)	0.5	0.8	0.3–1.6		
Immunosuppression	4 (3.6)	11 (9.8)	0.06	0.34	0.1–1.1		
Smoking							
Yes	21 (19)	11 (10)	0.05	2.1	0.9–4.6		
No	91 (81)	101 (90)					
Alcoholism							
Yes	14 (13)	8 (7)	0.17	1.8	0.7–4.6		
No	98 (87)	104 (93)					

SGSSS: *Sistema General de Seguridad Social en Salud* (General Social Security Health System); ESE: *empresa social del estado* (social state Company); HIV: human immunodeficiency virus; OR: odds ratio; 95%CI: 95% confidence interval.

## DISCUSSION

The study identified the factors of treatment abandonment in pulmonary tuberculosis, in which the variables considered correspond to aspects or factors related to adherence to tuberculosis treatment. Among the aspects are those related to the patient and the health services. The results showed that, among the patient-related factors in the abandonment of anti-tuberculosis treatment, are age, sex and vulnerable groups, which refer to drug addicts, prison inmates and street dwellers.

Regarding age and sex, the studies conducted by Arroyo et al.^3^ and Arenas et al.^4^ identified similar results regarding the presence of tuberculosis, with a greater presence in men than in women, at ages below 40 years old. However, they do not establish associations with abandonment, probably due to the different contexts and the specific characteristics of the populations studied^3^. In Colombia, a study investigating the barriers associated with adherence to shortened, strictly supervised tuberculosis treatment showed that people who use tobacco, alcohol and illegal drugs have, respectively, a 3.41 times greater chance of abandoning treatment than those who did not report using these types of substances^5^. This is in contrast to what was found in this study, where alcohol and tobacco consumption have no association, unlike the consumption of illegal drugs, showing a relationship present in vulnerable groups.

In addition, not having been tested for HIV is related to treatment abandonment (OR = 5.1), generating uncertainty in relation to the total number of people with a positive result. Authors cite in their studies that this comorbidity is related to the use of illicit drugs, which aggravates the situation by generating a poor level of follow-up to resistance^3,4^.

Regarding the abandonment factors related to the health service, there is evidence of the relationship between the absence of affiliation to the health system and treatment abandonment, similar to what was found in the study by Arenas et al.^4^, showing that the abandoning population does not have an affiliation system.

In our study, what could explain the relationship between abandonment and sociodemographic variables is the social crisis, in a city where the presence of extreme poverty and inequality makes it necessary for the economically active population to improve their daily income, which makes it difficult to adjust to the continuity of treatment^3^. Regarding the abandonment related to health services, it could have an implicit component related to the characteristics of the health system, malnutrition present in vulnerable populations, becoming risk factors for treatment abandonment^4^. Currently, in the covid-19 emergency, aspects related to the patient and health services may have a greater impact on treatment abandonment. Due to the limited evidence, future research is needed to establish the relationship between treatment abandonment in the public network and covid-19.

The limitations of this study are related to its retrospective nature and the use of secondary sources, that is, some variables were not available to fully explain the outcome; among them are marital status, occupation, means of transportation, place of diagnosis and persons with whom the patient lives. We consider that the strength of the study is that it is the first to determine the factors leading to abandonment of tuberculosis treatment in the public network. This entity is made up of 5 social enterprises of the state, responding to the need to organize health care for the poor and vulnerable population of the municipality in strata 1 and 2, in a city classified as high risk for tuberculosis, where the incidence of this disease is twice higher than that of Colombia.

In conclusion, we emphasize that the abandonment of tuberculosis treatment in the public network is driven by factors of the individual and of the health services, being determinant to evaluate the conditions that facilitate non-adherence, and distance the individual from the care provided in health institutions. However, it is necessary to rethink the strategies used so far in the control programs to allow vulnerable groups and the community in general to access care, since their living conditions represent a greater risk for increased tuberculosis transmission.
