# Supplementary material for: Factors of abandonment of tuberculosis treatment in the public health network
Source: Rev Saude Publica. 2023 Mar 15;57:8. doi: 10.11606/s1518-8787.2023057004454 (PMC10118406; doi:10.11606/s1518-8787.2023057004454)
Supplement: Translation

# Translation: Factores de abandono al tratamiento de la tuberculosis en la red pública de salud

**Authors:** Claudia Lorena Perlaza, Freiser Eceomo Cruz Mosquera, Luisa Maria Ramirez Murillo, Valentina Becerra Sepulveda, Cindy Dayan Córdoba Arenas

**Affiliations:** I Colombia Universidad Santiago de Cali. Facultad de Salud. Cali, Colombia

**Keywords:** Tuberculosis, prevención & control, Negativa del Paciente al Tratamiento, Antituberculosos, provisión & distribución, Barreras de Acceso a los Servicios de Salud

## Abstract

Este estudio determina los factores de abandono al tratamiento de la tuberculosis en la red pública de salud de Cali, Colombia, durante los años 2016 a 2018. Se realizó una investigación operativa de casos y controles en la que se incluyeron 224 pacientes con tuberculosis (112 abandonaron el tratamiento y 112 lograron completarlo). Se encuentra que el abandono del tratamiento para la tuberculosis está impulsado por factores relacionados con el individuo y los servicios de salud que facilitan la no adherencia y los alejan de la atención brindada en las instituciones médicas.

## INTRODUCCIÓN

La tuberculosis es una de las diez principales causas de muerte en el mundo, suscitada por Mycobacterium tuberculosis, y afecta a todos los grupos de edad. En 2019, se presentaron 10 millones de casos, con 1.4 millones de muertes. Esta enfermedad, con un diagnóstico oportuno y tratamiento ajustado al marco internacional del control de la tuberculosis, garantiza una sobrevida a las personas afectadas, con una tasa de éxito en el tratamiento del 57% a nivel mundial^1^.

La tuberculosis multirresistente, derivada de las reacciones del medicamento, y pérdida del seguimiento del tratamiento, representa medio millón de casos en todo el mundo. La administración interrumpida del fármaco es un factor determinante a esta resistencia, acaeciendo una amenaza para la salud pública y el sistema de salud, al bloquear la iniciativa de poner fin a la tuberculosis en todo el mundo, y al cumplimiento de los objetivos del desarrollo sostenible, donde se espera para el año 2023 una cobertura del 90% en el tratamiento^1^. Colombia, como país, aporta con una carga alta a la enfermedad de 19.000 casos de tuberculosis por año, y una tasa de incidencia de 35,8 por 100.000 habitantes^2^. Por lo anterior, el presente estudio tiene como objetivo determinar los factores de abandono al tratamiento de la tuberculosis en la red pública en Cali, Colombia, la cual responden por la atención de la población pobre y vulnerable, en una ciudad que tiene una tasa por encima de la nacional y contribuye a la mortalidad por tuberculosis en el mundo^1,2^.

## MÉTODOS

Investigación operativa con diseño de casos y controles, realizada en la red pública de s0alud de la ciudad de Santiago de Cali, con datos suministrados por el programa de tuberculosis de la Secretaría de Salud Pública Municipal. Se incluyeron pacientes mayores de 18 años, residentes en Cali, con diagnóstico de tuberculosis, que ingresaron al programa entre el 1 de enero de 2016 hasta el 31 de diciembre de 2018, y presentaron condición de abandono al tratamiento o pérdida en el seguimiento. Se consideró abandono no haber iniciado el tratamiento o interrupción de este durante un periodo igual o mayor a un mes.

Para calcular el tamaño de la muestra se usó la fórmula para estimación de proporciones, considerando: n = 721 sujetos con condición de abandono del tratamiento en el periodo de estudio, nivel de confianza de 95%, precisión de 5% y proporción esperada de 10% según datos de la Secretaría de Salud Pública Municipal de Cali, lo que sugirió una muestra mínima de 112 personas. Los participantes fueron seleccionados a partir de un muestreo probabilístico aleatorio simple y se compararon con 112 sujetos que sí completaron el tratamiento.

Con relación a las variables de estudio, se contemplaron variables sociodemográficas como edad, sexo, etnia, comuna de residencia y régimen de afiliación al sistema de salud; además, factores clínicos a saber: diagnóstico de VIH positivo, diabetes, malnutrición, tabaquismo, alcoholismo y farmacodependencia. Finalmente, se consideraron factores inherentes al servicio de salud como controles bacteriológicos realizados, meses de tratamiento recibido, programa de tuberculosis centralizado y equipo multidisciplinario de salud.

El análisis de los datos se realizó en el software SPSS versión 24. Para determinar la normalidad de las variables se utilizó la prueba de Kolmogorov Smirnov. Las variables categóricas se expresaron en frecuencia y porcentaje y las cuantitativas en promedio más desviación estándar. En el análisis univariado, se implementó chi-cuadrado en caso de diferencia de proporciones y T de Student para diferencia de medias, asumiendo un valor p < 0,05 como estadísticamente significativo. En el análisis bivariado, para establecer la asociación cruda entre las variables independientes y el abandono al tratamiento de la tuberculosis, se utilizaron tablas de contingencia a partir de las cuales se estimaron los *odds ratio* con sus respectivos intervalos de confianza del 95%. Finalmente se realizó una regresión logística binaria con bondad de ajuste de Hosmer-Lemeshow y método de selección por pasos hacia atrás “*backward*”, considerando como criterio de eliminación las variables con significancia mayor a 0.20.

La investigación siguió los lineamientos éticos de la Resolución 8430 del Ministerio de Salud de Colombia y de la Declaración del Helsinki. El protocolo contó con el aval ético de la Universidad Santiago de Cali y la autorización de la Secretaría de Salud Pública del Municipio de Cali.

## RESULTADOS

Se estudiaron 224 sujetos distribuidos de manera homogénea en el grupo de abandono y no abandono al tratamiento de la tuberculosis. En su mayoría, eran de sexo masculino (70%), mestizos (81%) y (56%) menores de 40 años. Con relación al estado de afiliación al Sistema General de Seguridad Social en Salud, el 77% eran del régimen subsidiado y el resto no estaban vinculados. Finalmente, en el modelo de regresión logística binaria se identificaron como factores asociados al abandono del tratamiento el sexo masculino (OR = 2.2; IC95% 1,1–4,5) la edad menor de 40 años (OR = 2,2; IC95% 1,2–4,2) hacer parte de una población vulnerable (OR = 3; IC95% 1,6–5,8) no estar asegurado (OR = 5,3; IC95% 2,2–12,3) y no haberse realizado la prueba para VIH (OR = 5,1; IC95% 1,5–17,8) (Tabla).

**Tabla:** Factores asociados al abandono de tratamiento de la tuberculosis.

Característica	Abandono n = 112	No abandono n = 112	p	OR crudo	IC95%	Exp (B)	IC95%
	
	n (%)	n (%)					
Sexo							
Masculino	86 (77)	70 (63)	0,02	1,9	1,1–3,5	2,2	1,1–4,5
Femenino	26 (23)	42 (37)					
Edad							
< 40 años	77 (69)	49 (44)	0,001	2,8	1,6–4,8	2,2	1,2–4,2
> 40 años	35 (31)	63 (56)					
Etnia							
Mestizo	95 (85)	88 (79)	0,22	1,5	0,7–2,0		
Otra	17 (15)	24 (21)					
Tipo de población							
Vulnerable	63 (56)	25 (22)	0,0001	4,4	2,5–7,9	3	1,6–5,8
Otras	49 (44)	85 (78)					
Régimen de afiliación al SGSSS							
No asegurado	41 (37)	10 (9)	0,0001	5,8	2,7–12,5	5,3	2,2–12,3
Subsidiado	71 (63)	102 (91)					
ESE centralizada							
No	38 (34)	40 (36)	0,7	1,08	0,6–1,8		
Sí	74 (66)	72 (64)					
Baciloscopia diagnóstica							
Negativa	8 (7)	4 (4)	0,4				
Una cruz	30 (27)	38 (34)					
Dos cruces	36 (32)	31 (27)					
Tres cruces	38 (34)	39 (35)					
Prueba de VIH							
No	15 (13)	5 (4)	0,019	3,3	1,1–9,4	5,1	1,5–17,8
Sí	97 (87)	107 (96)					
Antecedentes patológicos							
VIH	15 (13)	9 (8)	0,1	1,7	0,7–4,2		
Malnutrición	16 (14)	19 (17)	0,5	0,8	0,3–1,6		
Inmunosupresión	4 (3,6)	11 (9,8)	0,06	0,34	0,1–1,1		
Tabaquismo							
Sí	21 (19)	11 (10)	0,05	2,1	0,9–4,6		
No	91 (81)	101 (90)					
Alcoholismo							
Sí	14 (13)	8 (7)	0,17	1,8	0,7–4,6		
No	98 (87)	104 (93)					

SGSSS: Sistema General de Seguridad Social en Salud; ESE: empresa social del estado; VIH: virus de inmunodeficiencia humana; OR: *odds ratio*; IC95%: intervalo de confianza del 95%.

## DISCUSIÒN

El estudio identificó los factores de abandono al tratamiento en la tuberculosis pulmonar, donde las variables consideradas corresponden a aspectos o factores atingentes a la adherencia al tratamiento de la tuberculosis. Entre los aspectos están los relacionados con el paciente y los servicios de salud. Los resultados mostraron, que, entre los factores relacionados con el paciente en el abandono del tratamiento antituberculoso, se encuentran la edad, sexo y grupos vulnerables, que hacen referencia a la población farmacodependiente, carcelaria y habitante de calle.

En lo que respecta a la edad y el sexo, los estudios realizados por Arroyo et al.^3^, y Arenas et al.^4^, identificaron resultados similares, en lo concerniente a la presencia de tuberculosis; con mayor presencia en hombres que en mujeres, en edades por debajo de los 40 años. Sin embargo, no establecen asociaciones con el abandono, probablemente sea producto de los diferentes contextos, y de las características específicas de las poblaciones estudiadas^3^. En Colombia, un estudio donde se indagaron las barreras asociadas a la adherencia al tratamiento acortado, estrictamente supervisado de tuberculosis, evidenció que las personas que consumen tabaco, alcohol y drogas ilegales presentan, respectivamente, una oportunidad de 3.41 veces mayor de abandonar al tratamiento que las que no reportaron consumo de este tipo de sustancias^5^. Esto se opone a lo encontrado en este estudio, donde el consumo de alcohol y tabaco no tienen una asociación, a diferencia del consumo de drogas ilegales, donde se evidencia una relación presente en los grupos vulnerables.

Además, no haberse realizado prueba de VIH se relaciona con el abandono al tratamiento (OR = 5.1), generando incertidumbre frente al total de personas con un resultado positivo. Autores citan en sus estudios que esta comorbilidad se relaciona con el uso de drogas ilícitas, que agrava la situación, al generar un pobre nivel de seguimiento a la resistencia^3,4^.

En cuanto a los factores de abandono relacionados con el servicio de salud, queda en evidencia la relación de la ausencia de afiliación al sistema de salud y el abandono al tratamiento, similar a lo encontrado en el estudio de Arenas et al.^4^, donde la población que abandona no tiene un régimen de afiliación.

En nuestro estudio, lo que podría explicar la relación del abandono con variables sociodemográficas es la crisis social, en una ciudad, donde la presencia de pobreza extrema e inequidad hace necesario que la población económicamente activa requiera mejorar sus ingresos diarios, siendo difícil ajustarse a una continuidad en un tratamiento^3^. En lo que respecta al abandono relacionado a los servicios de salud, podría tener implícito un componente relacionado con las características del sistema de salud, la desnutrición presente en poblaciones vulnerables, que se convierten en factores de riesgo para el abandono del tratamiento^4^. En la actualidad, ante la emergencia por covid-19, los aspectos relacionados con el paciente y los servicios de salud pueden repercutir con mayor impacto en el abandono al tratamiento. Debido a la evidencia limitada, se hacen necesarias futuras investigaciones que permitan establecer la relación entre el abandono al tratamiento en la red pública y el covid-19.

Las limitaciones de este estudio se relacionan con su carácter retrospectivo y el uso de fuentes secundarias, es decir, no se contaba con algunas variables que permitieran explicar completamente el desenlace; entre ellas está el estado civil, la ocupación, el medio de transporte, el lugar del diagnóstico y las personas con quien convive. Consideramos que la fortaleza del estudio es ser el primero en determinar los factores de abandono al tratamiento de tuberculosis en la red pública. Entidad conformada por 5 empresas sociales del estado, que responden a la necesidad de organización de la atención en salud a la población pobre y vulnerable del municipio de los estratos 1 y 2, de una ciudad clasificada como de alto riesgo en tuberculosis, donde la incidencia de esta enfermedad es dos veces mayor que la de Colombia.

En conclusión, destacamos que el abandono del tratamiento para la tuberculosis en la red pública está impulsado por factores del individuo y de los servicios de salud, siendo determinante para evaluar las condiciones que facilitan la no adherencia, y lo alejen de la atención brindada en las instituciones de salud. No obstante, es necesario replantear estrategias utilizadas hasta el momento en los programas de control, que permitan que los grupos vulnerables y la comunidad en general accedan a la atención, pues sus condiciones de vida representan un mayor riesgo para el aumento de la transmisión de la tuberculosis.
